# Fish Collagen Surgical Compress Repairing Characteristics on Wound Healing Process In Vivo

**DOI:** 10.3390/md17010033

**Published:** 2019-01-08

**Authors:** Jingjing Chen, Kaili Gao, Shu Liu, Shujun Wang, Jeevithan Elango, Bin Bao, Jun Dong, Ning Liu, Wenhui Wu

**Affiliations:** 1 Department of Marine Bio-Pharmacology, College of Food Science and Technology, Shanghai Ocean University, Shanghai 201306, China; jingjingchen86@163.com (J.C.); m13052325756@163.com (K.G.); srijeevithan@gmail.com (J.E.); bbao@shou.edu.cn (B.B.); 2 Jiangsu Marine Resources Development Research Institute, Lianyungang 222000, China; jdliushu@163.com; 3 Co-Innovation Center of Jiangsu Marine Bio-Industry Technology, Huaihai Institute of Technology, Lianyungang, 222005, China; shujunwang86@163.com; 4 Shanghai Engineering Research Center of Aquatic-Product Processing & Preservation, Shanghai 201306, China; 5 German Rheumatism Research Center Berlin, 10117 Berlin, Germany; dong@drfz.de

**Keywords:** collagen, hydroxyproline, fibroblasts proliferation and differentiation, wound healing

## Abstract

The development of biomaterials with the potential to accelerate wound healing is a great challenge in biomedicine. In this study, four types of samples including pepsin soluble collagen sponge (PCS), acid soluble collagen sponge (ACS), bovine collagen electrospun I (BCE I) and bovine collagen electrospun II (BCE II) were used as wound dressing materials. We showed that the PCS, ACS, BCE I and BCE II treated rats increased the percentage of wound contraction, reduced the inflammatory infiltration, and accelerated the epithelization and healing. PCS, ACS, BCE I, and BCE II significantly enhanced the total protein and hydroxyproline level in rats. ACS could induce more fibroblasts proliferation and differentiation than PCS, however, both PCS and ACS had a lower effect than BCE I and BCE II. PCS, ACS, BCE I, and BCE II could regulate deposition of collagen, which led to excellent alignment in the wound healing process. There were similar effects on inducing the level of cytokines including EGF, FGF, and vascular endothelial marker CD31 among these four groups. Accordingly, this study disclosed that collagens (PCS and ACS) from tilapia skin and bovine collagen electrospun (BCE I and BCE II) have significant bioactivity and could accelerate wound healing rapidly and effectively in rat model.

## 1. Introduction

Skin trauma especially severe wound is a common clinical problem, and is more challenging to cure. The foremost aim for the treatment of skin defects is to rapidly restore the construction and function of the wound to the levels of normal tissue, involving acute and chronic inflammations, cell division, migration, and differentiation, regeneration and vascularization [1]. In recent years, the mechanism of wound healing properties of the biomaterials is becoming a research hotspot. Wound healing is a multifactorial process that is characterized by angiogenesis, collagen deposition, granulation tissue formation and re-epithelialization. All these phases involve complex biomolecular interactions among cells, soluble cytokines, adherence factors and chemokines. The clinical treatment of skin wound by traditional medicine has a long history from ancient times, however, the major drawback of traditional medicine dressing is less effective and prolonged treatment time. Many researchers focus on finding the new medical tissue engineering materials for wound healing. The medical tissue engineering materials can replace the damaged skin to provide temporary barrier function and avoid the wound being infected [2]. It provides a platform for cellular proliferation, adhesion and differentiation leading to the development of new functional tissues [3,4]. It can promote tissue repair, regeneration, and recovery to accelerate and complete wound healing [5]. Therefore, grafting tissue engineering material for the healing of a full-thickness wound is preferably a suitable model.

As a new tissue engineering material, collagen has good physical and mechanical properties [6], low immunogenicity [7,8,9], good biocompatibility, and biodegradability [10,11,12]. Due to minimal inflammation response, cytotoxicity effect, ability to promote cellular growth and good biocompatibility, collagen is the most promising skin substitute or wound dressing biomaterial [13]. Collagen can promote cellular adhesion and proliferation [14,15], collagen synthesis [16] and increase various growth factors [17], in order to accelerate wound-healing process. In earlier reports, collagen sponge from marine fish up-regulated the fibroblasts and keratinocytes growth, proliferation and wound healing potential in rat model [18]. Liane et al. [19] stated that neurotensin-loaded collagen dressings significantly reduced inflammatory cytokine expression, increased fibroblast migration, enhanced collagen I/III expression and deposition. Tian et al. [20] also reported that electrospun tilapia collagen nanofibers could significantly promote the proliferation of human keratinocytes (HaCaTs), stimulate epidermal differentiation and facilitate rat skin regeneration. All these findings claim that collagen is an excellent biomaterial to be used in wound healing purpose.

Biomimetic environment is also essential for tissue regeneration. Electrospun nanofibrous matrix has been proved to be very effective in skin regeneration because of its superiority features including adjustable diameters, porosity, mimic the structure and function of native extracellular matrix (ECM) and high surface-to-volume ratio, which are beneficial for cell adhesion and proliferation [21]. If collagen could be prepared as nanofibers by electrospinning, it might be helpful for its future application.

Our earlier study showed that collagens from Tilapia skin (PCS, ACS) have significant biocompatibility and can be absorbed and degraded by tissues [22]. Bovine collagen electrospun has been confirmed significant biocompatibility and no cytotoxicity. In continuation to our earlier research [23,24,25,26], in vivo wound healing properties of tilapia collagen sponges and bovine collagen electrospun were studied in rat models in order to evaluate its mechanism on accelerating wound healing properties.

## 2. Results

### 2.1. Macroscopic Observation of the Wounds

**Figure 1:**
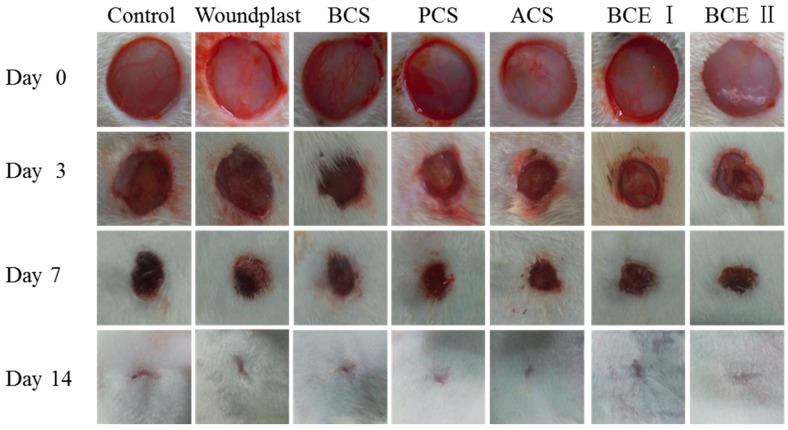
The different stages of wound healing in rats treated with collagens. BCS—bovine collagen sponge, PCS—pepsin soluble collagen sponge, ACS—acid soluble collagen sponge, BCE I—bovine collagen electrospun I, BCE II—bovine collagen electrospun II.

Representative images of wound healing process at different time intervals across all the experimental groups are shown in Figure 1. Collagen-treated groups showed faster wound healing process (complete healing after 14 days from wound incision) compared to control group, in detail, the wound regions were covered with epidermis and the wound areas were closed. With the time extending, wound area of each group decreased gradually. On day 3, each group showed different degree of collagen absorption and wound areas of BCS, PCS, ACS, BCE I and BCE II treated groups were obviously smaller than control and woundplast groups; on day 7, the wounds of the groups treated with BCS, PCS, ACS, BCE I, and BCE II contracted further and scabbed, the wounds of control and woundplast groups had only a small amount of granulation tissue; on day 14, dried blood fall-off from the wound and the wounds healed completely. The wounds which were treated with PCS, ACS, BCE I, and BCE II were smooth and no pigmentation. Interestingly, ACS and PCS treated groups exhibited faster healing than control, woundplast, BCS and BCE I groups, however, BCE II treated group had better healing ability than other groups.

### 2.2. The Quantification of Aggregate Protein at the Wound Site

An adequate supply with proteins is necessary for consistent wound healing. Therefore, protein content can be used to evaluate the conditions of wound healing [27]. Total protein expression at the wound site of all groups in the excision wound model is shown in Table 1. On day 3 and 7, protein content of the experimental groups was higher than the control and the woundplast groups (*p* < 0.05), and the protein content was high in BCE I and BCE II treated groups compared to other groups on day 7 (*p* < 0.05). On day 14, protein content of the experimental groups was significantly higher than control group (*p* < 0.05). Protein content of ACS treated group was significantly higher than BCS, BCE I, and BCE II treated groups (*p* < 0.05).

### 2.3. Hydroxyproline (Hyp) Content at the Wound Site

Hyp is a specific component of the protein collagen. Therefore, Hyp content might be used as an indicator to determine collagen deposition to measure the speed of wound healing [28]. The significant increase of Hyp in collagen treated group implied faster rate of wound healing process than control group (*p* < 0.05). Indeed, collagen is a major protein of the extracellular matrix and it ultimately contributes to wound healing [29]. Hyp content at the wound site of all groups in the excision wound model is shown in Table 2. Hyp content was higher in ACS and BCE II treated groups (5.87 ± 0.42 mg/g, 5.66 ± 0.12 mg/g tissue) on day 3 and in PCS, ACS, BCE II treated groups on day 7 (*p* < 0.05). On day 14, PCS and ACS treated groups had high Hyp content (7.32 ± 0.43 mg/g, 7.41± 0.42 mg/g tissue) than control and woundplast treated groups.

### 2.4. Histopathological Examination

**Figure 2:**
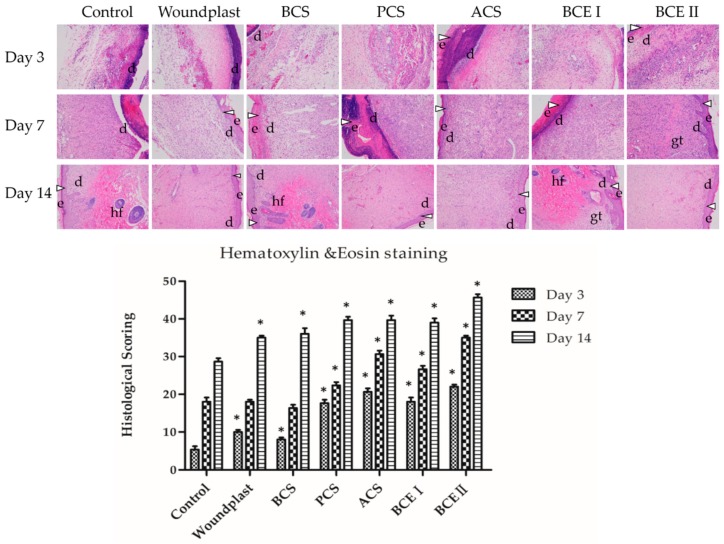
Histological analysis of H&E stained wounded tissues (Magnification, ×100) with histological scoring. The alphabetic letters e, d, gt and hf represent epidermis, dermis, granulation tissue, hair follicle, respectively. (Each bar represents the mean ± SD. * *p* < 0.05: significantly different from the control group.).

In control group, a large number of inflammatory cells appeared in the wound than collagen-treated groups on day 3 (Figure 2). Granulation tissue and fibroblasts activity were more pronounced in collagen-treated groups compared to control group. A large number of inflammatory cells, blood vessels, small amount of collagen fibers and fibroblasts were seen in woundplast and BCS groups. Fibroblast cells were important in the wound site and predominant collagen expression could be seen in PCS, ACS, BCE I, and BCE II treated groups. On day 7, healed regions of the wounds were covered by epithelial tissue with significant fibroblast proliferation, collagen deposition and granulation tissue formation. However, the granulation tissue organization and vascularization in unhealed regions of collagen-treated groups were notably different from the control group. Collagen-treated groups developed collagen deposition and vascularization than the control group. The H&E staining revealed that in collagen-treated wound on day 7, cutaneous appendages like hair, hair follicles and sebaceous glands began to appear, which signify the formation of epidermal layer. A large number of inflammatory cells and collagen fibers and fibroblasts were observed in control group. A large number of fibroblasts and collagen were seen in woundplast and BCS treated groups and skin appendages and skin tissues were gradually formed in collagen treated groups. More fibroblasts and collagen and less inflammatory cells were seen in PCS, ACS, BCE I, and BCE II treated groups than the control group. On day 14, a sufficient number of fully formed skin adnexal and epithelial tissues were present in collagen-treated groups. PCS, ACS, BCE I, and BCE II treated groups showed well-formed stratified epithelial layer, granulation tissue formation and collagen deposition in healed regions than the control, woundplast and BCS groups. As shown in Table 3, a scoring system was used to quantify the pathological results of the H&E stained samples. In this scoring system five criteria were scored. An increasing score denotes the better wound healing. The histological scoring result showed that the PCS, ACS, BCE I, and BCE II treated groups had better wound healing than the control group.

### 2.5. Collagen Promotes the Expression of EGF, FGF, and CD31 in the Wounds

**Figure 3:**
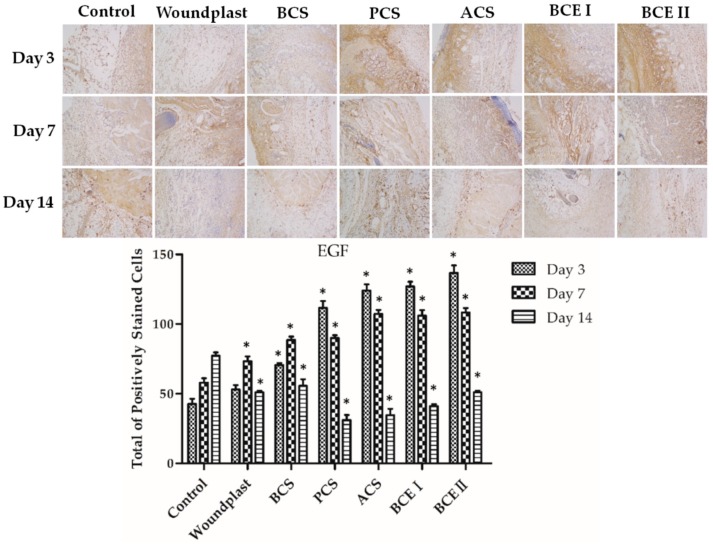
Immunohistochemical analysis of EGF expression in wounded tissues (Magnification, ×100); the histogram shows the total of positively stained cells of EGF in the dermal tissue per group. (Each bar represents the mean ± SD. * *p* < 0.05: significantly different from the control group.).

**Figure 4:**
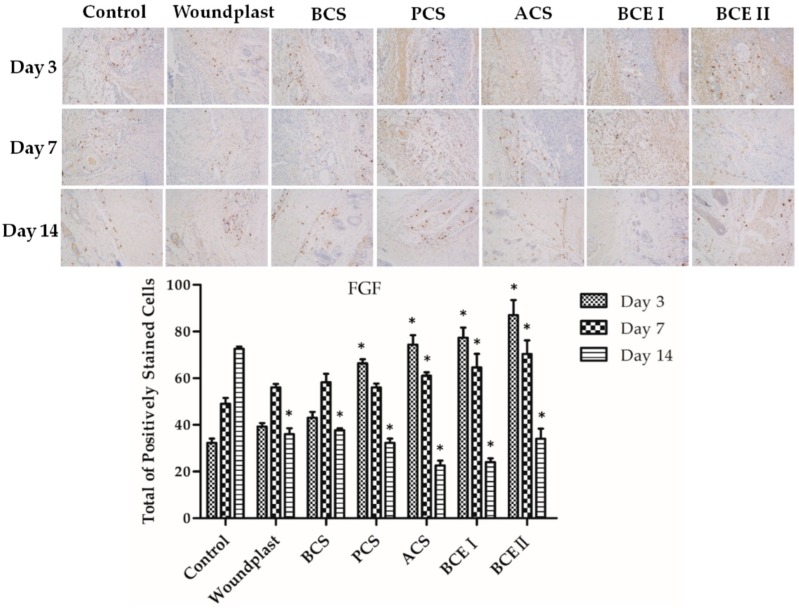
Immunohistochemical analysis of fibroblast growth factor (FGF) expression in wounded tissues (Magnification, ×100); the histogram shows the total of positively stained cells of FGF in the dermal tissue per group. (Each bar represents the mean ± SD * *p* < 0.05: significantly different from the control group.

**Figure 5:**
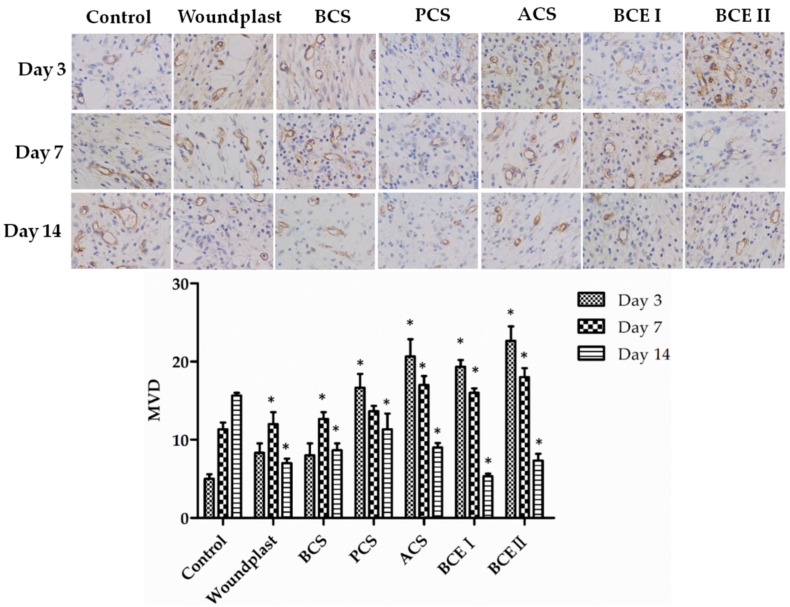
Immunohistochemical analysis of CD31 expression in wounded tissues (Magnification, ×200). The histogram summarizes the microvessel density (MVD), which was determined by immunohistochemical staining for CD31. (Each bar represents the mean ± SD. * *p* < 0.05: significantly different from the control group.).

Immuno-histochemical analysis of the reconstituted tissue was shown in Figure 3, Figure 4 and Figure 5. EGF stands for epidermal growth factor, which can promote proliferation and differentiation of keratinocyte. EGF can induce fibroblasts proliferation and collagen synthesis, resulting in epithelization. Fibroblast growth factor (FGF) can accelerate migration and proliferation of fibroblast, and vascularization. EGF and FGF play an important role in the wound healing process. Prolonging the treated time, the expression of EGF and FGF in control group showed an increasing trend, the expression of EGF and FGF in woundplast and BCS groups increased in the beginning and then decreased, and the expression of EGF and FGF in PCS, ACS, BCE I, and BCE II treated groups showed a decreasing trend. On day 3 and 7, the expression of EGF and FGF in PCS, ACS, BCE I, and BCE II treated groups was higher than control, woundplast and BCS groups. CD31 is a platelet endothelial cells adhesion molecule-1, stands for vessel proliferation. The relative quantity and distribution of CD31 in the construct-treated wound bed is important in wound healing. Brown granules were positive signal in Figure 3. The expression of CD31 showed similar trends same as EGF and FGF of collagen treated groups.

## 3. Discussion

As the structural and functional component of dermal extracellular matrix, collagen plays a vital role in wound healing process [18,30,31,32]. In the present study, PCS, ACS, and electrospun bovine skin collagen nanofibers were successfully fabricated. Due to its unique perforated structure, controllable fiber diameter (50 nm–5 μm), large surface area, high porosity and unique biological property [33], PCS, ACS and BCE I, BCE II were examined by using the full-thickness wound model in SD rats and the total protein, hydroxyproline content, H&E, and immunohistochemical examinations were assessed with control group. In full-thickness wounds in SD rats, PCS, ACS, BCE I and BCE II treated groups revealed significantly higher wound healing ability, total protein and hydroxyproline content, fibroblasts proliferation and collagen synthesis when compared to control, BCS and woundplast groups.

Based on the earlier findings, the possible reasons for accelerating wound healing of PCS, ACS, BCE I, and BCE II were due to its unique structures and the high porosity of the matrix, which could induce fibroblasts proliferation and collagen synthesis, and play crucial roles in re-epithelialization and vascularization in the wound healing process [34,35]. In addition, PCS, ACS, BCE I, and BCE II exhibited good biocompatibility to support the adhesion and proliferation of fibroblasts that lead the deposition and maturation of collagen [9]. Collagen could be completely degraded and absorbed by the wound that helps to formation of fibroblasts and collagen fibers over time in epidermal tissue and therefore, the wounds were replaced with regenerated dermis.

Histopathological and immunohistochemical examinations indicate that PCS, ACS, BCE I, BCE II had a positive effect on neovascularization, inducing fibroblasts proliferation, collagen synthesis, re-epithelialization and regeneration of skin appendages, and consequently led to an increased wound healing ability compared with control, BCS and woundplast groups. Besides, PCS, ACS, BCE I and BCE II were similar to normal skin because of smooth surface along with loose collagen fiber. PCS, ACS, BCE I, and BCE II could provide a proper microenvironment for fibroblasts attachment in skin due to its three-dimensional features and high porosity.

Vascular endothelial marker CD31, growth factors EGF and FGF are involved in wound healing process [36]. It has been confirmed that PCS, ACS, BCE I, and BCE II significantly induce EGF and FGF expression, which can promote proliferation and differentiation of fibroblasts and keratinocytes. EGF can induce keratinocytes proliferation to impel re-epithelialization of the wound. As chemotactic agent and mitogenic agent of fibroblasts, EGF can promote the proliferation and differentiation of fibroblasts to synthesize collagen. The increased expression of CD31 and FGF revealed the vascularization and wound healing properties of collagens.

In this work, PCS and ACS groups revealed significantly better wound healing ability than woundplast and control groups, slightly higher than BCS group, which might be due to the three-dimensional features and high porosity of PCS and ACS than BCS. However, ACS had a more positive effect than PCS, which was similar to previous report [22]. There was no much difference between BCE I and BCE II treated groups. However, there was less inflammatory cells in BCE II treated group than BCE I, which may be related to the bactericidal effect of chitosan. These effects were probably owed to the biomimetic structure, unique biological property and high porosity of the collagen nanofibers.

## 4. Materials and Methods

### 4.1. Materials

Bovine collagen electrospun (BCE) was prepared by Department of Textile Materials College, Donghua University, Shanghai, China, and its biocompatibility and cytotoxicity were confirmed. Bovine collagen sponge (BCS) prepared with polyethylene oxide (PEO) (BCE I) and PEO with chitosan (BCE II) were compared with tilapia collagens. The weight ratio of bovine collagen, PEO and chitosan was 150:17:2. Tilapia skin collagens were prepared by Shanghai Ocean University, Shanghai, China. Briefly, Pepsin soluble collagen sponge (PCS) and acid soluble collagen sponge (ACS) from Tilapia skin were prepared as per our previous method [22]. Both collagens were composed of two α-chains and a β-chain and characterized as type-I collagen [22]. BCS was kindly provided by HaoHai Biological Technology, Shanghai, China, which was characterized as type-I collagen. Woundplast was purchased from Johnson & Johnson (Shanghai, China) and medical gauze was procured from Shanghai Health Materials Factory Co. Ltd., Shanghai, China.

SD rats were purchased from Shanghai Slac Laboratory Animal Co. Ltd., Shanghai, China. Animal study protocols and procedures were approved by the Shanghai Ocean University institutional animal care and use committee (Permit Number: SHOU-DW-2018-054). All methods were employed in accordance with the relevant guidelines and regulations of Scientific and Ethical Care and Use of Laboratory Animals of Shanghai Ocean University.

### 4.2. Skin Wound Healing in SD Rats

The wound healing experiment was performed as follows: female rats (body weight 200–250 g) were maintained in a pathogen free environment and fed a standard diet. 63 rats were injected intraperitoneal with sodium pentobarbital. Two full-thickness wounds of size 1 cm in diameter were created on the dorsum of SD rats which were 1.5 cm apart from the spine. These wounds were covered with medical gauze, woundplast, BCS, PCS, ACS, BCE I, and BCE II (*n* = 6) to avoid infections. Control group was served without any treatment (only medical gauze). Medical adhesive tape was used to attach the dressings in the wounds. Dressing change was done every two days and rats were kept in individual cages. On days 3, 7, and 14 after surgery, the morphology of the wounds was examined. The skin wounds of animals were photographed and subsequently, the rats were euthanized. Wound tissues were removed by sacrificing three rats each from all groups periodically on the 3rd, 7th, and 14th days of post wound creation and the granulation tissues formed were collected.

### 4.3. Determination of Total Protein and Hydroxyproline Content

Skin tissue (~90 ± 10 mg) was taken from the wound for determination of total protein and hydroxyproline content. The harvested skin samples collected on days 3, 7, and 14 were washed with ice-cold saline and dried by filter paper. They were then used to determine hydroxyproline and total protein level in specimen [37]. The HYP content in tissue samples was determined using a Tissue hydroxyproline kit, as per the manufacturer’s instruction (JianChen Gene Company, Nanjing, China). The healed skin tissues (*n* = 6) were harvested and cut into pieces and then incubated with tissue lysis buffer for 20 min at 95 °C and homogenized. The tubes were centrifuged (13,000 rpm) at 4 °C and supernatant was collected. Total protein concentration was evaluated using a total protein kit (JianChen Gene Company, Nanjing, China) according to the manufacturer’s instructions. The average value was taken from the triplicate readings.

### 4.4. Histopathological Examination

Harvested wounds together with the surrounding skins were used for the histological evaluation. The harvested samples collected on days 3, 7, and 14 were fixed in 10% buffered formalin solution for 24 h. The tissues were embedded in paraffin and sectioned into 5 μm thick slices for histopathological examination by hematoxylin and eosin (H&E) staining method. Then they were studied by a routine light microscope. The criterion that was studied in histopathological sections consisted of re-epithelialization, collagen deposition, fibroblast content, revascularizations, and inflammatory cells. Analysis of stained skin sections was performed by an experienced pathologist.

### 4.5. Immuno-Histochemical Examinations

The skin tissues including the wound site were excised and fixed in 10% buffered formalin for more than 24 h, then embedded in paraffin and cut into 5 μm thick slices. After deparaffinization and rehydration, antigen unmasking was performed as follows: the endogenous peroxidase of randomly selected section was inactivated by incubation with 3% hydrogen peroxide/methanol solution at 37 °C for 30 min. The slices were washed three times with PBS for 5 min each wash. In order to recover antigen, these sections were put into 10 mM citrate buffer solution (pH 6.0) and heated at 95 °C for 15 min, and then cool down at room temperature, followed by washing three times with PBS for 5 min each wash. The non-specific binding sites were blocked with 5% goat serum (Gibco, 16210072) for 10 min at 37 °C. After the redundant liquid was discarded, the sections were incubated with the following primary antibodies, respectively: anti-EGF antibody, anti-FGF antibody, and anti-CD31 antibody at 4 °C overnight and washed three times with PBS for 5 min, followed by incubation with biotinylated goat anti-rabbit secondary antibody kit (Santa Cruz, Shanghai, China) at 37 °C for 30 min, and then incubated with streptavidin-HRP for 30 min. The slides were dyeing with a DAB (3,3’-diaminobenzidine) solution, and then counterstained with hematoxylin and following by dehydration with sequential ethanol for sealing and microscope observation. FGF and EGF positive cells were analyzed from three identical areas in the dermal tissue per rat wound tissue section and analyzed for the statistical significance. Individual micro-vessels were counted at 200× magnification (0.152 mm^2^/field). For each section, three areas were selected from the vascularity of the wound tissues [38].

### 4.6. Statistical Analysis

The values were expressed as the mean ± standard deviation (SD). Statistically significant differences (*p* < 0.05) among the different groups were evaluated using Student’s *t*-test and one-way analysis of variance (ANOVA) with Tukey’s post hoc multiple comparison test. All of the statistical analyses were performed using SPSS 17.0 software.

## 5. Conclusions

In this study, PCS, ACS, BCE I, and BCE II were successfully fabricated and evaluated for its utility as dermal substitute. PCS, ACS, BCE I, and BCE II treatment increased the wound healing ability, fibroblasts proliferation, collagen synthesis, re-epithelialization and dermal reconstitution in vivo that owed to the biomimetic structure and high porosity of the collagen nanofibers. This study indicated that PCS, ACS, BCE I, and BCE II could accelerate wound healing rapidly and effectively. The overall results of this study suggest that collagen from tilapia and electrospun bovine collagen nanofibers can be used as a promising dermal substitute to treat severe wounds.

## Figures and Tables

**Table 1:** The effect of each group on total protein content in wound area tissues ($\bar{x}$ ± *s*, *n* = 6).

Groups	Total Protein (mg/mL)		
	3 Days	7 Days	14 Days
Control	7.93 ± 0.6 ^a^	8.03 ± 1.1 ^a^	9.15 ± 0.7 ^a^
Woundplast	8.12 ± 0.6 ^b^	9.15 ± 0.4 ^b^	10.3 ± 0.7 ^cd^
BCS	8.61 ± 0.4 ^bc^	9.52 ± 1.4 ^bc^	9.94 ± 1.1 ^b^
PCS	8.48 ± 0.8 ^bc^	9.58 ± 0.8 ^bc^	11.7 ± 1.3 ^bc^
ACS	8.65 ± 0.4 ^c^	9.66 ± 1.3 ^b^	13.3 ± 0.8 ^c^
BCE I	8.79 ± 0.9 ^c^	10.2 ± 0.8 ^c^	11.4 ± 0.4 ^bd^
BCE II	8.85 ± 0.5 ^c^	10.5 ± 0.7 ^c^	11.3 ± 0.5 ^bd^

Note: Different superscript alphabets in each column represent statistical significance (*p* < 0.05).

**Table 2:** The effect of each group on the Hydroxyproline content in wound area tissues ($\bar{x}$ ± *s*, *n* = 6).

Groups	Hydroxyproline Content (mg/g Wet Skin)		
	3 Days	7 Days	14 Days
Control	5.26 ± 0.33 ^a^	6.12 ± 0.29 ^a^	6.79 ± 0.39 ^a^
Woundplast	5.46 ± 0.41 ^ab^	6.38 ± 0.49 ^a^	7.29 ± 0.18 ^ab^
BCS	5.60 ± 0.24 ^ab^	6.29 ± 0.36 ^a^	7.02 ± 0.39 ^ab^
PCS	5.47 ± 0.36 ^ab^	6.55 ± 0.44 ^b^	7.32 ± 0.43 ^b^
ACS	5.87 ± 0.42 ^b^	6.65 ± 0.34 ^b^	7.41 ± 0.42 ^b^
BCE I	5.42 ± 0.25 ^ab^	6.42 ± 0.35 ^a^	6.77 ± 0.33 ^a^
BCE II	5.66 ± 0.12 ^b^	6.68 ± 0.54 ^b^	7.22 ± 0.27 ^ab^

Note: Different superscript alphabets in each column represent statistical significance (*p* < 0.05).

**Table 3:** Histological evaluation of wound tissue in all groups by H&E staining.

	0	1–3	4–6	7–9
Inflammatory cells	Abundant	Moderate	Scant	Rarely
Fibroblast content	None	Scant	Moderate	Abundant
Re-epithelialization	None	Partial	Thin	Complete
Collagen deposition	None	Scant	Moderate	Abundant
Revascularizations	None	Scant	Moderate	Abundant
